# Academy calls on South Africans to vaccinate

**DOI:** 10.4102/safp.v63i1.5396

**Published:** 2021-10-25

**Authors:** Robert Mash

**Affiliations:** 1 Division of Family Medicine and Primary Care, Faculty of Medicine and Health Sciences, Stellenbosch University, Cape Town, South Africa; 2 South African Academy of Family Physicians, Cape Town, South Africa

Coronavirus disease 2019 (COVID-19) vaccine hesitancy in South Africa has been fuelled by conspiracy theories and misinformation. Unfortunately, some of this misinformation has come from health professionals, the most notable recent example being Dr Susan Vosloo, a cardiothoracic surgeon in Cape Town. However, family physicians have also been seen to promote unproven treatments such as ivermectin and even nebulised colloidal silver.

Our approach to COVID-19 should be led by science and an evidence-based approach as the science evolves. Vaccines have been shown to prevent hospitalisation, severe disease and death from COVID-19. In the United Kingdom (UK) (based on data from the Office for National Statistics), the high levels of vaccination led to a dramatic reduction in deaths between the second and third waves of COVID-19.^1^ In the second wave the number of average daily cases reached 33 per 100 000 population, and in the third wave, with the more infectious Delta variant, 34 per 100 000 – so very similar numbers of cases. However, in the second wave the average daily deaths reached 36 per 10 million and in the third wave only 2.1 per 10 million. It is clear, therefore, that while vaccines do not fully prevent transmission, they do save lives.

Four different types of vaccines have been developed based on messenger ribonucleic acid, viral vectors, viral proteins and inactivated virus. In South Africa, the AstraZeneca viral vector vaccine was effective against the ancestral strains of COVID-19 in the first wave but not against the beta variant, which drove the second wave of infection in South Africa.^1^ This is why the vaccine was not implemented in South Africa. Healthcare workers have been vaccinated with the viral vector Johnson and Johnson vaccine, which is efficacious against ancestral strains (72%) and the beta variant (64%), although data on the delta variant is still awaited. The messenger ribonucleic acid Pfizer BioNTech vaccine has been shown effective against ancestral strains (92%), the beta variant (100% in one South African cohort) and the delta variant (64% – 87%). The Johnson and Johnson vaccine is given as one dose, while Pfizer requires two doses, and the full effect is only seen after the second dose.

Vaccines may cause minor side effects in the 24–48 h after injection, such as pain and redness at the injection site, headache, mild fever, chills or myalgia.^2^ Anaphylaxis can occur but is extremely rare. The South African Health Products Regulatory Authority has investigated all deaths that occurred after vaccination and has not yet found any evidence to link them to the vaccination.^3^ In South Africa vaccination is also recommended in pregnant women after the first trimester.^4^

It is likely that COVID-19 is here to stay, and the future of this pandemic may depend on what additional variants emerge to drive new waves of infection. We hope that the current vaccines will continue to be effective against future variants, although this is not guaranteed. In the scenario of ‘vaccine escape’, booster doses may be needed to protect against new mutations. The likelihood of new mutations emerging is, however, related to the size of the pool of infection and ongoing replication of the virus. Evidence is beginning to emerge that people with human immunodeficiency virus (HIV), who remain immunocompromised, have difficulty clearing the virus from the body and may be particularly prone to incubating new mutations.^5^

Getting vaccinated is the best approach to protecting ourselves against hospitalisation and death from COVID-19. It will also help to reduce symptoms, transmission and the viral load in those infected. Although death from COVID-19 is more likely in certain groups, such as the elderly, men, and those with diabetes or hypertension;^6^ no age group is entirely risk free. The South African Academy of Family Physicians therefore urges all South Africans to become vaccinated to protect themselves and those around them.

As of the beginning of August we had vaccinated 8 million people in South Africa, a remarkable achievement in a short time period.^1^ We need to increase our rate of vaccination even more, to try and protect our population ahead of a possible fourth wave in the summer period. Africa, unfortunately, is the continent with the least vaccine coverage, and many low- and middle-income countries have struggled to secure a supply of vaccine, while many high-income countries have bought up more than their share. Canada, for example, bought 10 doses for every citizen, and Australia and the UK 5 doses for every citizen.^1^ We must also fight against such inequity in access to the vaccines and ensure that our government has secured sufficient supplies.

In closing, therefore, I urge our membership to speak out publicly and encourage your communities to get vaccinated, to challenge misinformation and conspiracy theories, and to assist with vaccinating our population. Watch COVID-19 vaccine video at: https://youtu.be/KVnNeZnSOwY.^7^
